# Supplementary material for: β-Catenin interacts with canonical RBPs including MSI2 to associate with a Wnt signalling mRNA network in myeloid leukaemia cells
Source: Oncogene. 2025 Apr 30;44(29):2490–503. doi: 10.1038/s41388-025-03415-y (PMC12256266; doi:10.1038/s41388-025-03415-y)
Supplement: Supplementary file 1 — Supplementary material [file 41388_2025_3415_MOESM1_ESM.docx]

**Supplementary Table S1. Clinical characteristics of AML patient diagnostic/relapse samples used in this study**.

| **Patient no.** | **Age (at diagnosis)** | **Sex** | **WBC count (x10^9^/L)** | **Sample type** | **Secondary disease (Y/N)** | **Genetic information** | **Other clinical information** |
| --- | --- | --- | --- | --- | --- | --- | --- |
| 1 | 4 | F | 2.6 | BM | N | Normal karyotype, NPM1^+^ (exon 12), FLT3^-^ | MRD neg. CD13+, CD33+, CD34+, CD117+, MPO+ |
| 2 | n/a | n/a | n/a | n/a | n/a | n/a | n/a |
| 3 | n/a | n/a | n/a | n/a | n/a | n/a | n/a |
| 4 | 14 | F | 7.6 | BM | N | MLL rearrangement. Karyotype: 46,XX,ins(10;11)(q11.2;q23.1q23.3).ish ins(10;11)?inv(11)(q23.3)(5’MLL+)(q23.1)(3’MLL+) | BMT for high-risk AML. CD33+, MPO+, CD34-, CD117+, TdT+, CD64+, CD11c+, CD15+, CD11b+, NG2+ |
| 5 | n/a | n/a | >200 | PB | Y | n/a | Post-allogeneic transplant. M0/1 (previously diagnosed with M3 10 years previous) |
| 6 | 6 | M | 70.7 | n/a | Y | n/a | Secondary to Ewings Sarcoma. Myelomonocytic morphology. Deceased. |
| 7 | 5 | M | 1.6 | BM | N | n/a | M5. Deceased. |
| 8 | 16 | F | 20.6 | BM | N | Normal karyotype 46,XX[20] | CD33+, MPO+, CD34+, CD117+, CD13+, CD14-, CD7+, CD45 weak, CD11c+, TdT- |
| 9 | 10 | F | n/a | BM | n/a | n/a | Deceased |
| 10 | 76 | F | 389 | LP | N | Normal karyotype, NPM1^+^, FLT3^+^ | N/A |
| 11 | 7 | M | 3.5 | BM | N | t(8;21)(q22;q22) RUNX-RUNX1T1  45,X,-Y,t(8;21)(q22;q22)[9]/46,XY[1] | 8% myeloid blasts present (CD13+, CD33+, CD34+, CD117+, MPO+) |
| 12 | 7 | F | n/a | BM | N | MPAL, 46XX, del5q, abnormal 21 | n/a |
| 13 | 64 | F | 13.3 | BM | N | Normal karyotype | AML with underlying MDS like changes |
| 14 | 7mo | F | 168.6 | BM | N | Karyotype: 47,XX,+21[5]/46,XX[5] nuc ish(CBFA2T3,GLIS2)X3(CBFA2T3 con GLIS2x2)[92/150]  FISH showed no evidence of CBFB or RUNX1-RUNX1T1 rearrangement however, an additional copy of RUNX1 was found.  33+, CD34+, CD117+, MPO+, DR-, CD13+ | BMT 24/12/20 due to high risk genetics.  Alive, in remission.  Chromosome and FISH analysis showed an inv(16)(p13.3q24.3) [CBFA2T3-GLIS2] rearrangement and trisomy for chromosome 21. This is consistent with a diagnosis of AML and the CBFA2T3-GLIS2 rearrangement is a poor risk finding according to the MyeChild 01 protocol (received Gemtuzumab as part of Myechild trial treatment.) |
| 15 | 17 | M | n/a | BM | Y | n/a | Post-BMT for AML following 2 relapses. Deceased |
| 16 | 4 | M | 3.2 | BM | N | t(10;11)(p11.2;q23) KMT2A-MLLT10. Cytogenetically cryptic. KMT2A ex8-MLLT10 ex9 or KMT2A ex9-MLLT10 ex10 fusion detected. NPM1^-^FLT3^-^ | CD13-, CD33+, CD34-, CD117+/-, CD11c+, CD64+, CD14-, NG2+. High risk cytogenetics. BMT. MRD neg post course 1+2. |
| 17 | n/a | n/a | n/a | n/a | n/a | n/a | n/a |
| 18 | 15 | F | 6.5 | BM | N | MLL (KMT2A) rearrangement, t(10;11)(p11-p14,q23), MLL-MLLT10 | MRD detected post treatment course 1 |
| 19 | 2 | M | 2.4 | BM | N | t(9;11)(p22;q23), t(11;21)(q23;q8) | n/a |
| 20 | 7 | F | 34.4 | BM | Y | MLL rearrangement t(9;11) | M5a morphology. BMT following relapse. Deceased |

BM = Bone marrow

PB = Peripheral blood

LP = Leukapheresis

MRD = Minimal residual disease

BMT = Bone marrow transplant

AML= Acute myeloid leukemia

MDS= Myelodysplastic syndrome

MPAL= Mixed phenotype acute leukemia

MLL = *Mixed-lineage leukemia*

NPM1 = *Nucleophosmin*

FLT3 = *Fms-like tyrosine kinase 3*

RUNX1 = *Runt-related transcription factor 1*

GATA2 = *GATA Binding Protein 2*

**Supplementary Table S2 - Forward and reverse primer sequences used for RT-qPCR.**

| **Primer** | **5’ -> 3’ sequence** |
| --- | --- |
| *ACTB* Fw | TTGTTACAGGAAGTCCCTTGCC |
| *ACTB* Rv | ATGCTATCACCTCCCCTGTGTG |
| *GAPDH* Fw | ACAGTCAGCCGCATCTTCTT |
| *GAPDH* Rv | ACGACCAAATCCGTTGACTC |
| *AMER1* Fw | AGTACCCGTGAACAAAGAGCA |
| *AMER1* Rv | AGGCAGTACAGATACCCTTC |
| *BCL9L* Fw | TGAACCTGAACGTGCAGATGA |
| *BCL9L* Rv | CCCTGGTTGGGAAACTGTG |
| *AXIN2* Fw | TTGGCTACTCCGTAAAGTTTTGGT |
| *AXIN2* Rv | TACACTCCTTATTGGGCGATCA |
| *LEF1* Fw | AGAACACCCCGATGACGGA |
| *LEF1* Rv | GGCATCATTATGTACCCGGAAT |
| *TCF7L2* Fw | AGAAACGAATCAAAACAGCTCCT |
| *TCF7L2* Rv | CGGGATTTGTCTCGGAAACTT |
| *MYB* Fw | GAAAGCGTCACTTGGGGAAAA |
| *MYB* Rv | TGTTCGATTCGGGAGATAATTGG |
| *MYC* Fw | AGCGACTCTGAGGAGGAA |
| *MYC* Rv | CCAGCAGAAGGTGATCCA |
| *RNA18SN1* Fw | CTCAACACGGGAAACCTCAC |
| *RNA18SN1* Rv | CGCTCCACCAACTAAGAACG |

**Supplementary Table S3 – Lentiviral plasmids used in this study for transgene expression.**

| **Target gene** | **Expression type** | **Vector type** | **Supplier** |
| --- | --- | --- | --- |
| Non-targeting | shRNA control | pLKO.1-puro Non-Mammalian shRNA Control Plasmid  SHC002 | Merck MISSON® |
| *MSI2* | shRNA (#1) | pLKO_TRCN0000062808 | Merck MISSON® |
| *MSI2* | shRNA (#2) | pLKO_TRCN0000062809 | Merck MISSON® |
| *CTNNB1* | shRNA (#1) | pLKO_TRCN0000314920 | Merck MISSON® |
| *CTNNB1* | shRNA (#2) | pLKO_TRCN0000314921 | Merck MISSON® |
| Empty vector control | Ectopic | pLV[Exp]-Puro-EF1A>ORF_91bp  (VB160723-1006snj) | VectorBuilder |
| *MSI2* | Ectopic | pLV[Exp]-Puro-EF1A>hMSI2[NM_138962.4]  Vector ID:VB230612-1123ebn | VectorBuilder |
| Empty vector control | Ectopic | pLV[Exp]-mCherry-EF1A>ORF_91bp  (VB230711-1149jnd) | VectorBuilder |
| *MSI2* | Ectopic | pLV[Exp]-mCherry-EF1A>hMSI2[NM_138962.4]  Vector ID:VB230612-1125btf | VectorBuilder |
| Non-targeting | shRNA control | pLV[shRNA]-EGFP-U6>Scramble[shRNA#2] (Vector ID:VB230321-1431mhe) | VectorBuilder |
| *LEF1* | shRNA (#1) | pLV[shRNA]-EGFP-U6>hLEF1  TRCN0000418104 | VectorBuilder |
| *LEF1* | shRNA (#2) | pLV[shRNA]-EGFP-U6>hLEF1  TRCN0000428355 | VectorBuilder |


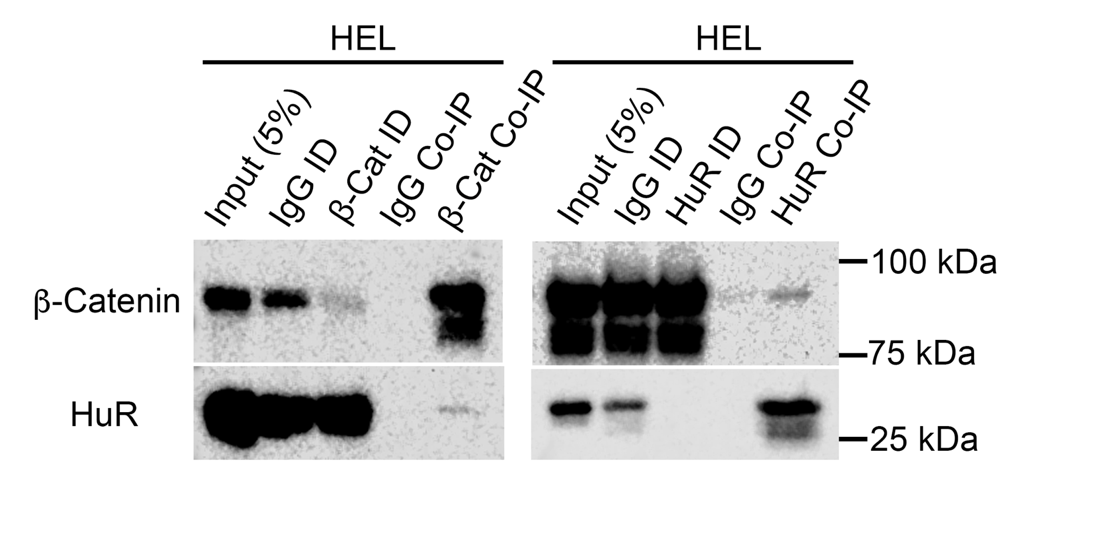


**Supplementary Figure S1. HuR and β-Catenin interact in AML cells.** Immunoblots showing the level of HuR (*ELAVL1*) protein present in β-catenin Co-IP, and reciprocally the level of β-catenin present in HuR Co-IP from whole cell lysates derived from HEL cells.


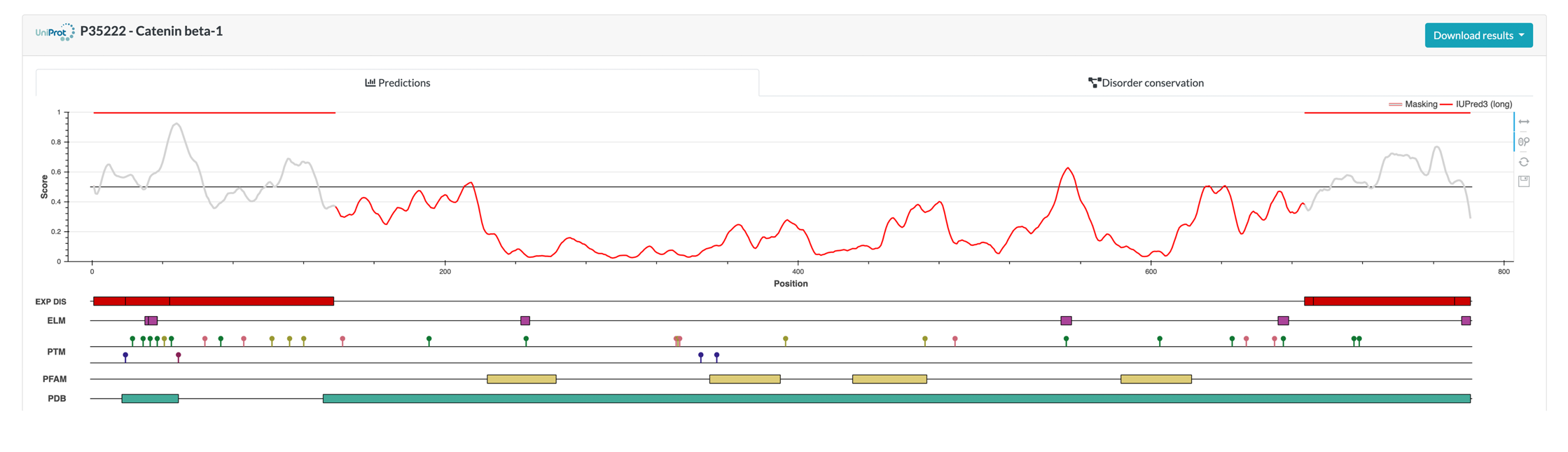


**Supplementary Figure S2. β-Catenin intrinsically disordered regions (IDRs).** Amino acid residue map from IUPred3 server predicting the most likely location within the β-catenin peptide for IDRs which are the N- and C-termini with values between high confidence values between 0.5-1 (Erdos, G. et al. *Nuc Acids Res*, 2021).


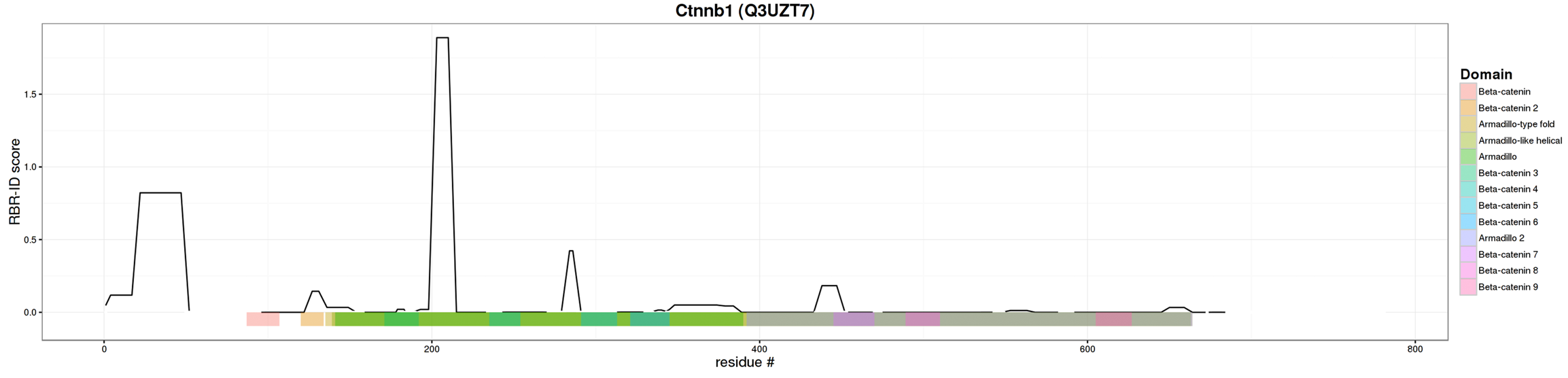


**Supplementary Figure S3. β-Catenin central armadillo domains predict RNA binding.** Amino acid residue map from Bonasio Lab RBR-ID browser v0.3 showing likely RNA-binding regions (RBR) in the ARM domain (seq: TMQNTNDVETAR, RBR-ID score: 1.89) and N-terminus (seq:

AAVSHWQQQSYLDSGIHSGATTTAPSLSGK, RBR-ID score: 0.82) of β-catenin from nuclei of murine embryonic stem cells following RBP screen (He, C. *et al*, *Mol Cell*, 2016).


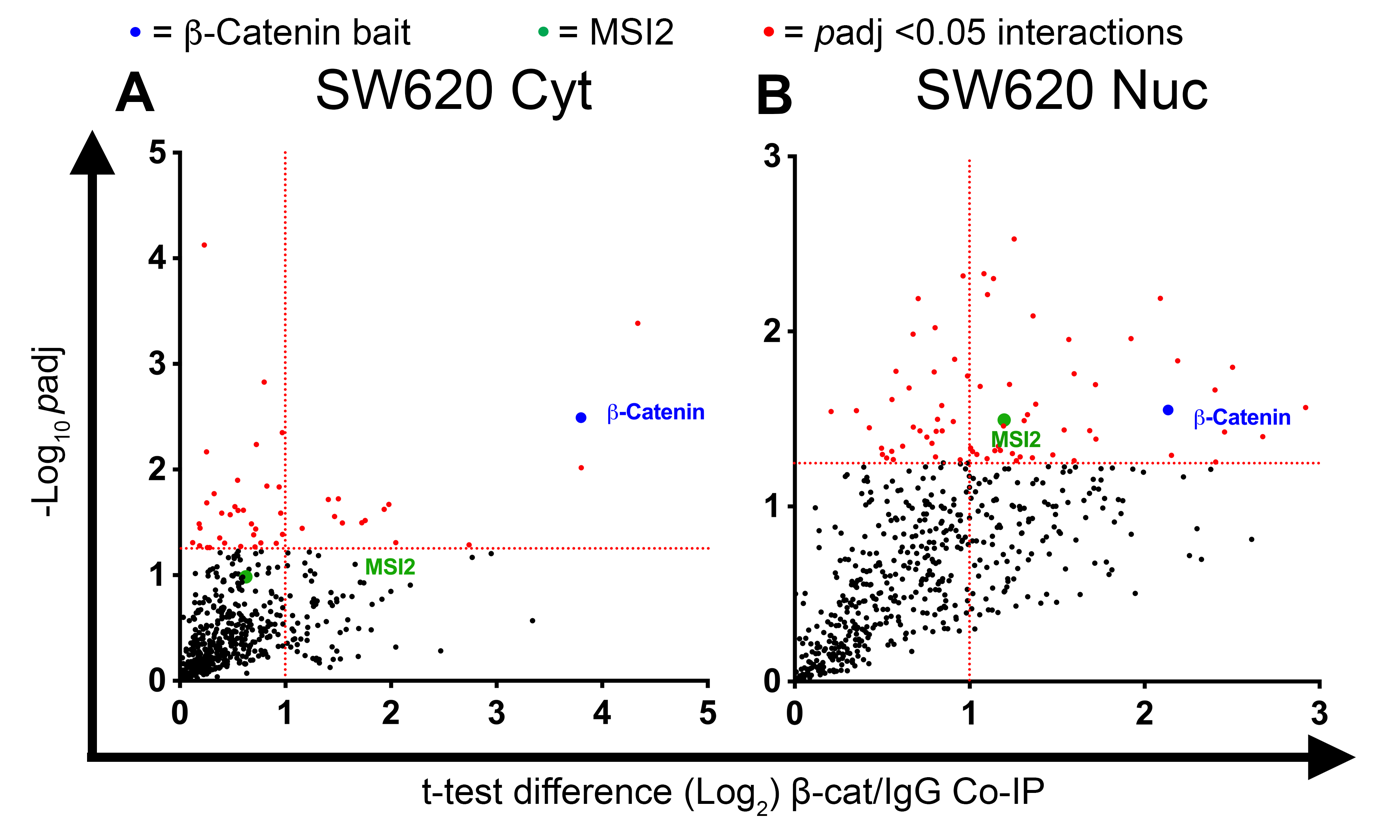


**Supplementary Figure S4. Proteomics analyses reveal β-catenin:MSI2 interaction in colorectal cancer cells.** Scatter plots showing β-catenin protein interactions detected in SW620 cytosolic and nuclear fractions. Vertical dashed red line indicates the threshold for 2-fold change in protein binding at log_2_ (=1) relative to IgG co-immunoprecipitation. Horizontal red line represents *P*=0.05 on log_10_ scale (=1.3). Highlighted red dots indicate interactions where p<0.05. Remaining black dots represent other proteins detected in the MS analysis, green dot highlights MSI2 detection. Fold change values less than 0 are not shown because these likely represent contaminants.


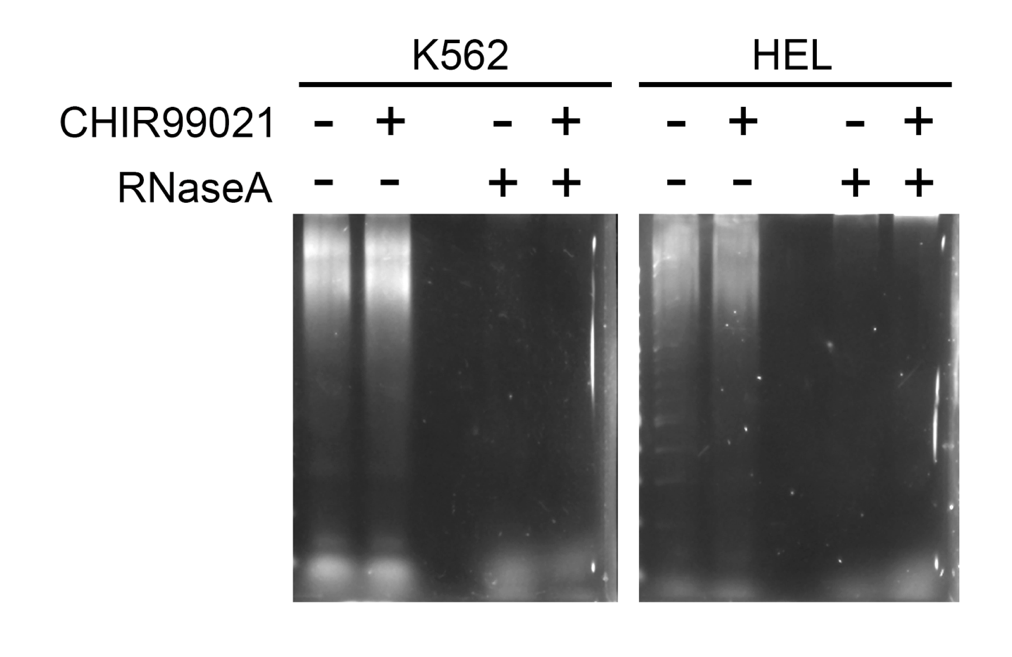


**Supplementary Figure S5. RNA digestion through RNaseA treatment.** Agarose gel electrophoresis showing the stability of total RNA in K562 and HEL lysates treated overnight +/-5mM CHIR99021 (or respective DMSO control) and +/- 20 mg/mL RNaseA.

**
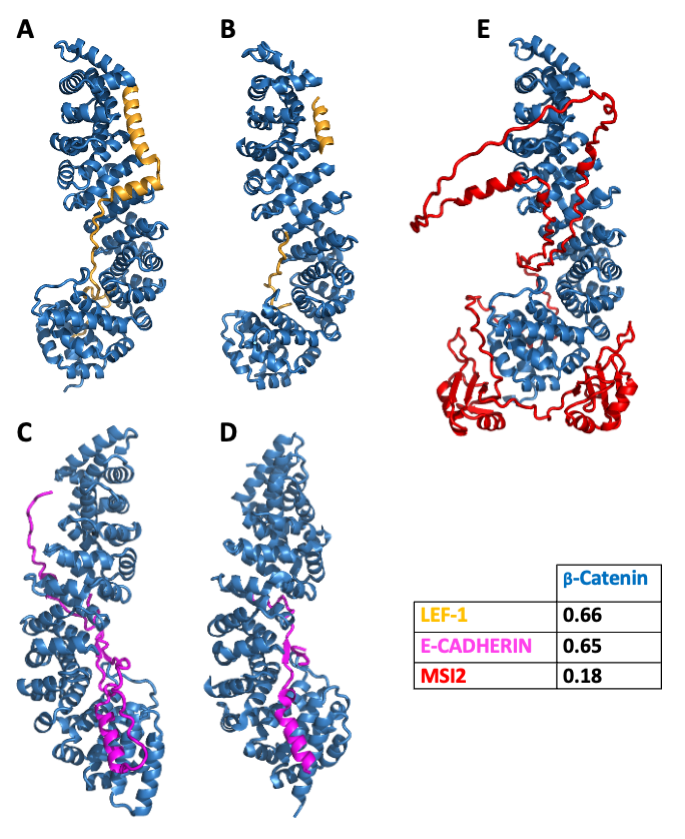
**

**Supplementary Figure S6. AlphaFold3 prediction of β-catenin protein-protein interactions.** Cartoon representations of β-catenin (blue), LEF-1 (yellow), E-cadherin (magenta), MSI2 (red). Alphafold3 prediction model **(A)** and crystal structure (3OUW) **(B)** of the β-catenin-LEF-1 complex. Alphafold3 prediction model **(C)** and crystal structure (4R1O) **(D)** of the β-catenin-E-cadherin complex. Alphafold3 prediction model of the β-catenin-MSI2 complex **(E)**. Table shows the AlphaFold3 predicted interface TM-score (iptm), a measure of the confidence in the predicted interfaces, for each complex.


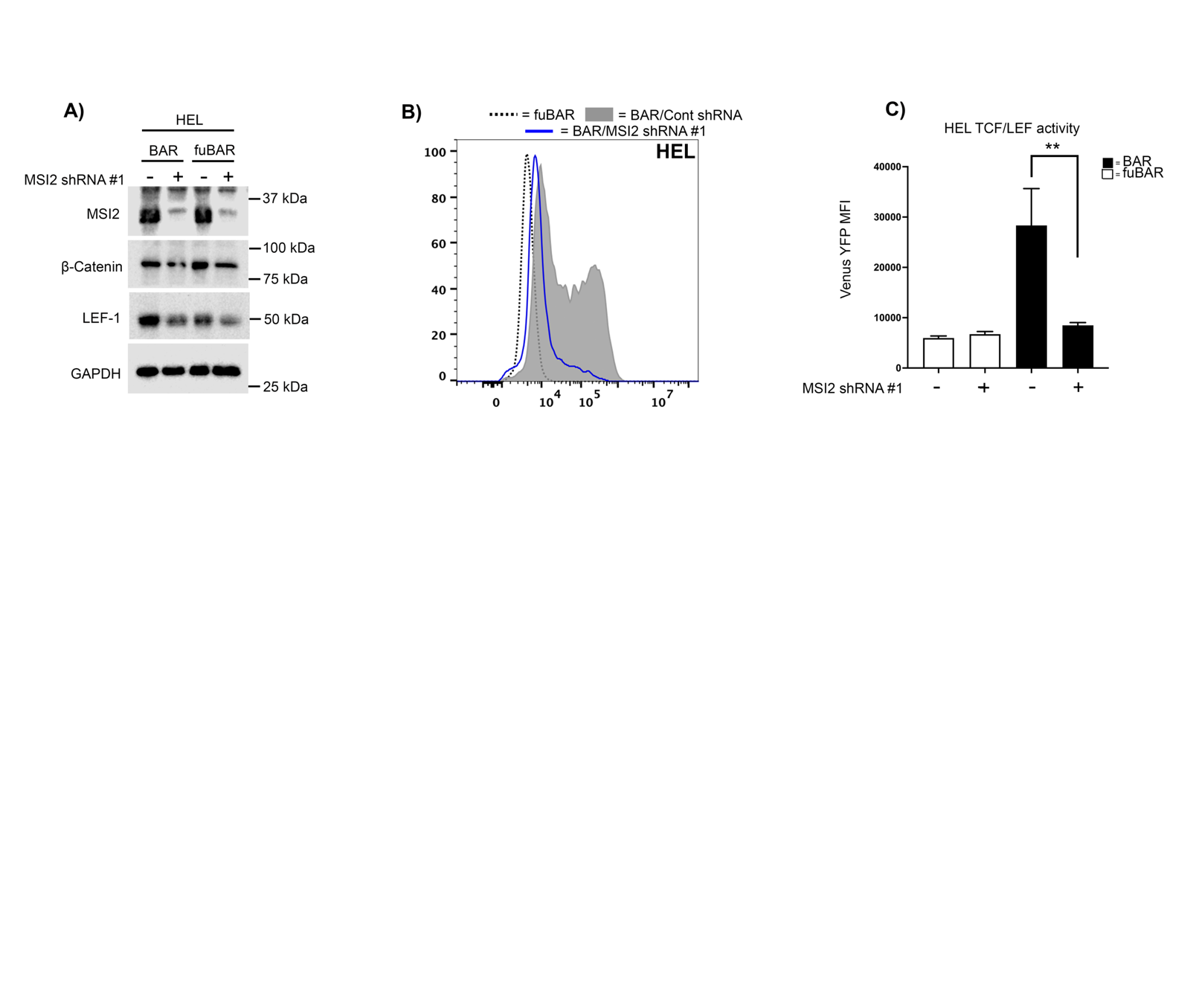


**Supplementary Figure S7. MSI2 knockdown impairs Wnt signalling output in HEL cells. A)** Immunoblots showing total MSI2, β-catenin and LEF-1 level in HEL cells (Wnt reporter BAR and fuBAR variants) harbouring MSI2 shRNA or non-targeting shRNA control. GAPDH indicates protein loading. **B)** Representative flow cytometric histograms showing YFP intensity BAR or negative control fuBAR HEL cells +/- MSI2 shRNA following treatment with 5mM CHIR99021 overnight. The fuBAR (dashed), non-targeting control shRNA (grey filled), and MSI2 shRNA (blue) histograms are shown. **C)** Summary bar graphs showing the median YFP fluorescence intensity (MFI) generated from the BAR/fuBAR in HEL +/- MSI2 shRNA with +/- 5mM CHIR99021. All data represents mean ± 1 s.d (*n* = 3). Statistical analysis is denoted by ***p*<0.005 as deduced by a student’s paired t-test.


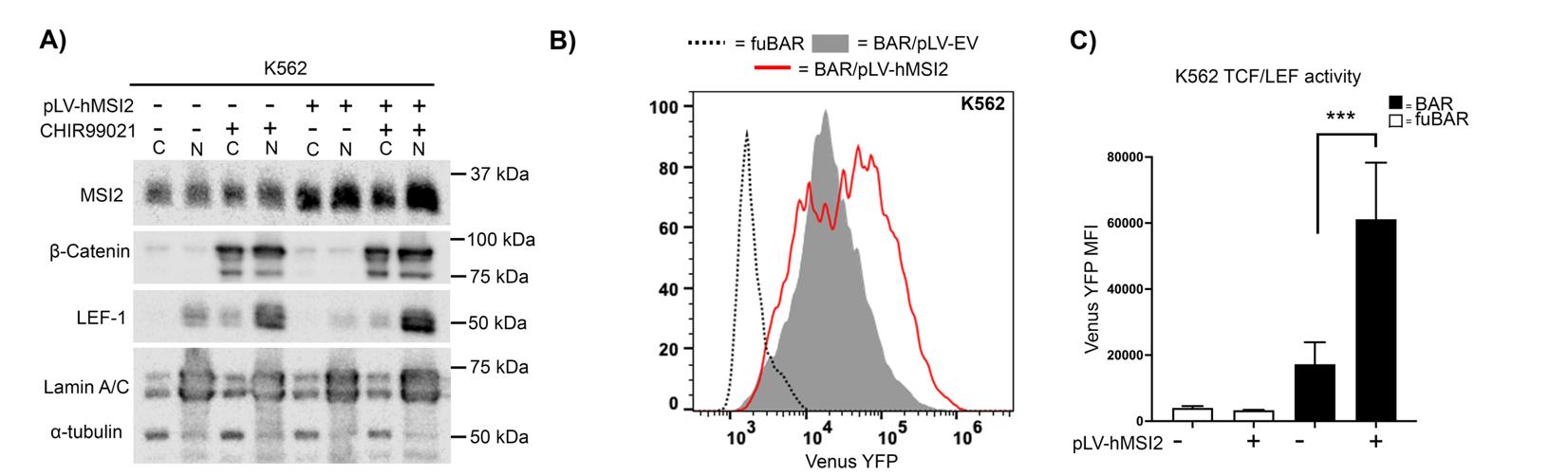


**Supplementary Figure S8. MSI2 overexpression enhances Wnt signalling in K562 cells. A)** Immunoblots showing total β-catenin, LEF-1 and MSI2 subcellular localization in K562 cells lentivirally transduced with ectopic human MSI2 (pLV-hMSI2) +/- 5mM CHIR99021. Lamin A/C and α-tubulin indicate the purity/loading of the nuclear (N) and cytosol (C) fractions, respectively. **B)** Representative flow cytometric histograms showing intensity of the TCF-dependent expression of Venus YFP from the BAR reporter, or negative fuBAR control in K562 cells +/- ectopic MSI2 OE shRNA following treatment with 5mM CHIR99021 overnight. The fuBAR (dashed), non-targeting control shRNA (grey filled), and MSI2 OE shRNA (red) histograms are shown. **C)** Summary bar graphs showing the median fluorescence intensity (MFI) generated from the BAR/fuBAR in K562 +/- MSI2 OE shRNA with +/- 5mM CHIR99021. All data represents mean ± 1 s.d (*n* = 3). Statistical anlaysis is denoted by ****p*<0.0005 and as deduced by a paired t-test.


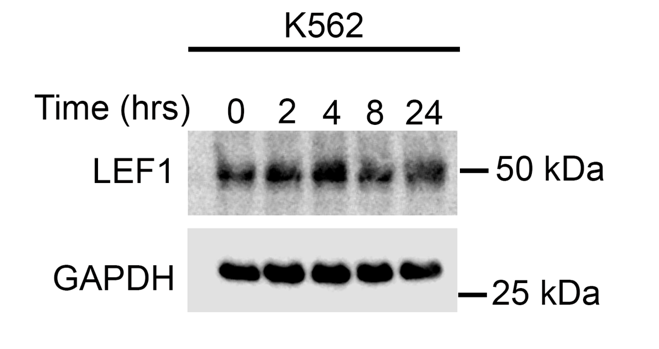


**Supplementary Figure S9. The LEF-1 peptide has a long half-life.** Immunoblot showing the total level of LEF-1 protein following treatment with 5μg/ml of Actinomycin D exposure for 2, 4, 8 and 24 hours. GAPDH is used to indicate protein loading.


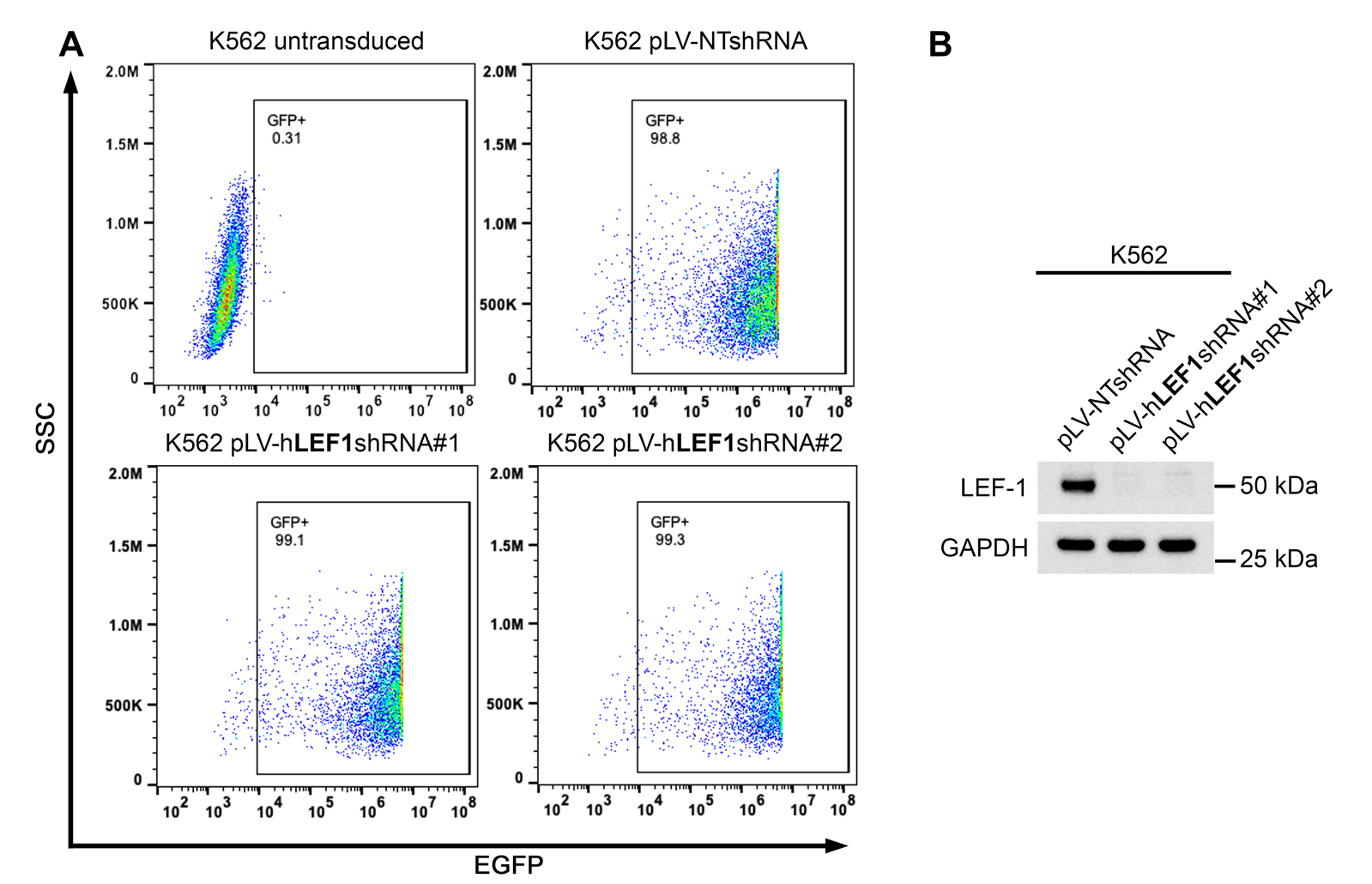


**Supplementary Figure S10. LEF-1 shRNAs are functional. A)** Representative flow cytometric pseudocolour plots demonstrating enhanced green fluorescent protein (EGFP) positivity percentage in K562 cells 3 days post-lentiviral transduction with pLV plasmids containing non-targeting (NT) control shRNA or two unique LEF1 shRNA sequences. The untransduced parental K562 line is also shown to demonstrate where the threshold for GFP positivity was set. **B)** Immunoblot showing the total level of LEF-1 protein following lentiviral transduction of K562 cells with pLV plasmid containing LEF1 shRNA#1 and shRNA#2 sequences. GAPDH is used to indicate protein loading.
